# Molecular defects in primary ciliary dyskinesia are associated with agenesis of the frontal and sphenoid paranasal sinuses and chronic rhinosinusitis

**DOI:** 10.3389/fmolb.2023.1258374

**Published:** 2023-10-03

**Authors:** Andre Schramm, Johanna Raidt, Anika Gross, Maik Böhmer, Achim Georg Beule, Heymut Omran

**Affiliations:** ^1^ Department of General Pediatrics, University Hospital Muenster, Muenster, Germany; ^2^ Clinic for Radiology, University Hospital Muenster, Muenster, Germany; ^3^ Department of Otorhinolaryngology, Head and Neck Surgery, University Hospital Muenster, Muenster, Germany

**Keywords:** ciliopathy, PCD, genotype phenotype correlation, sinusitis, health related quality of life

## Abstract

**Background:** Primary ciliary dyskinesia (PCD; MIM 242650) is a rare genetic disorder characterized by malfunction of the motile cilia resulting in reduced mucociliary clearance of the airways. Together with recurring infections of the lower respiratory tract, chronic rhinosinusitis (CRS) is a hallmark symptom of PCD. Data on genotype–phenotype correlations in the upper airways are scarce.

**Materials and methods:** We investigated the prevalence, radiologic severity, and impact on health-related quality of life (HrQoL) of CRS in 58 individuals with genetically confirmed PCD. Subgroup analysis was performed according to the predicted ultrastructural phenotype based on genetic findings.

**Results:** Among 58 individuals harboring pathogenic variants in 22 distinct genes associated with PCD, all were diagnosed with CRS, and 47% underwent sinus surgery. A total of 36 individuals answered a German-adapted version of the 20-item Sinonasal Outcome Test (SNOT-20-GAV) with a mean score of 35.8 ± 17, indicating a remarkably reduced HrQoL. Paranasal sinus imaging of 36 individuals showed moderate-to-severe opacification with an elevated Lund–Mackay Score (LMS) of 10.2 ± 4.4. Bilateral agenesis of frontal sinus (19%) and sphenoid sinus (9.5%) was a frequent finding in individuals aged 16 years or older. Subgroup analysis for predicted ultrastructural phenotypes did not identify differences in HrQoL, extent of sinus opacification, or frequency of aplastic paranasal sinuses.

**Conclusion:** PCD is strongly associated with CRS. The high burden of disease is indicated by decreased HrQoL. Therefore, the upper airways of PCD individuals should be evaluated and managed by ear–nose–throat (ENT) specialists. Genetically determined PCD groups with predicted abnormal *versus* (near) normal ultrastructure did not differ in disease severity. Further studies are needed to gain evidence-based knowledge of the phenotype and management of upper airway manifestations in PCD. In addition, individuals with agenesis of the frontal and sphenoid paranasal sinuses and chronic respiratory symptoms should be considered for a diagnostic evaluation of PCD.

## 1 Introduction

Motile cilia have a highly ordered ultrastructure with a canonical 9 + 2 microtubule-based organization (Wallmeier et al., 2020). Multiple motile cilia lining the cell surface of different tissues beat in a coordinated slow backward and fast forward stroke in order to clean the airways. The upper airways also comprise the nose, paranasal sinuses, and middle ears. Primary ciliary dyskinesia (PCD; ORPHA:244) is a rare genetic disorder affecting the function of motile cilia (Mirra et al., 2017). This results in impaired mucociliary clearance and a wide spectrum of clinical symptoms and manifestations in various organ systems (Werner et al., 2015; Paff et al., 2021). To date, pathogenic variants in more than 50 genes are related to PCD (Wallmeier et al., 2020). The severity and course of the disease differ based on the genotypes (Paff et al., 2021). Some of these ciliary defects cause ultrastructural changes that are detectable by transmission electron microscopy (TEM), whereas others do not (Shoemark et al., 2020). Therefore, the diagnostics of PCD are complex, and several tests, including measurement of nasal nitric oxide, high-speed video microscopy of vital ciliated epithelial cells (i.e., collected by nasal brushes), immunofluorescence analyses, TEM, and genetic analyses, are needed (Lucas et al., 2017a).

There is no cure for PCD, and its management focuses on symptomatic treatment (Paff et al., 2021). As good clinical evidence is missing, the management of PCD is often adopted to experiences in the more frequent and well-investigated respiratory disease cystic fibrosis (CF) (Lucas et al., 2017b; Marthin et al., 2021). Both diseases show an impaired mucociliary clearance leading to bacterial colonization, recurring/chronic infections, and progressive changes of the upper and lower airways (i.e., bronchiectasis) (Marthin et al., 2021). However, the pathophysiology is different, and some highly effective therapies in CF, such as inhalation of recombinant human dornase alfa, do not benefit individuals with PCD (Paff et al., 2021).

The majority of individuals with PCD suffer from symptoms related to the paranasal sinuses, and half of these patients fulfil the formal criteria for chronic rhinosinusitis (CRS) (Sommer et al., 2011; Bhatt et al., 2019). Zawawi et al. demonstrated that nasal congestion (83%) and nasal discharge (77%) were the most frequent upper airway symptoms in a cohort of children with PCD and that sinonasal disease can lead to a decrease in health-related quality of life (Zawawi et al., 2021). Pifferi *et al.* investigated the sense of smell in individuals with PCD and showed an inverse correlation between loss of smell and radiologic grading of CRS. In the cohort, sense of smell was reduced in PCD individuals with abnormal ciliary ultrastructure compared to PCD individuals with normal ultrastructure due to pathogenic variants in *DNAH11* (Pifferi et al., 2018). However, data on CRS in individuals with PCD remain limited, including detailed genotype–phenotype correlation.

In this study, we investigated the impact of CRS in a large cohort of individuals with genetically confirmed PCD on health-related quality of life (HrQoL), and we graded CRS severity based on radiologic imaging and the Lund–Mackay scoring system. We grouped PCD individuals based on genetic defects that are predicted to cause either (near) normal or abnormal ciliary ultrastructure for genotype–phenotype correlation.

## 2 Materials and methods

### 2.1 Study design and population

We performed prospective analyses of HrQoL and retrospective analyses of computed tomography (CT) and magnetic resonance imaging (MRI) scans of paranasal sinuses in individuals with a genetically confirmed diagnosis of PCD.

The study population included individuals from the PCD cohort of the University Hospital Muenster, Germany (https://pcdregistry.uni-muenster.de/), who were referred between 02/2011 and 09/2021 for PCD diagnostics. Only patients with genetically confirmed PCD and available paranasal sinus imaging and/or SNOT-20-GAV were included. Each participant or their legal guardian(s) gave written informed consent prior to participation. The study was approved by the local ethics committee of the Westphalian Wilhelms-University of Muenster (Muenster, Germany; AZ 2011-270-f-S). PCD diagnosis was confirmed following the ERS diagnostic guideline (Lucas et al., 2017a).

Mutations in many different genes can cause PCD due to marked genetic heterogeneity. We divided the individuals into two groups depending on the mutated genes, as described previously (Raidt et al., 2022; Kinghorn et al., 2023). One gene group predicted abnormal axonemal ultrastructure detectable by TEM. This group comprised i) outer dynein arm (ODA)-, ii) combined inner dynein arm (IDA)/ODA-, and iii) microtubular disorganization and IDA defects (Shoemark et al., 2020). The other gene group predicted normal or near-normal ultrastructural phenotypes [referred to as *(near) normal ultrastructure*] of the respiratory ciliary axonemes (Raidt et al., 2022; Kinghorn et al., 2023). Please refer to Table 1 for the different gene groups.

**TABLE 1:** Genetic findings and predicted ultrastructure for each individual. Abbreviation: ID, Identification number; Var. class, Variant classification; 4 likely pathogenic, 5 pathogenic.

ID	Allele 1			Allele 2			Predicted ciliary ultrastructure	
	Mutation	Protein level	Var. Class	Mutation	Protein level	Var. Class	(Near) normal	Abnormal
1-15	ZMYND10(NM_015896.4):c.47T>G	(p.Val16Gly)	5	ZMYND10(NM_015896.4):c.47T>G	(p.Val16Gly)	5		X
1-16	ODAD1(NM_144577.4):c.742G>A	(p.Ala248Thr)	5	ODAD1(NM_144577.4):c.742G>A	(p.Ala248Thr)	5		X
1-18	ZMYND10(NM_015896.4):c.47T>G	(p.Val16Gly)	5	ZMYND10(NM_015896.4):c.490dup	(p.Gln164ProfsTer19)	4		X
1-19	DNAI1(NM_012144.4):c.48+2dup	(p.?)	5	DNAI1(NM_012144.4):c.912C>G	(p.Tyr304Ter)	5		X
1-25	DNAH5(NM_001369.3):c.2710G>T	(p.Glu904Ter)	5	DNAH5(NM_001369.3):c.2710G>T	(p.Glu904Ter)	5		X
1-28	DNAH11(NM_001277115.2):c.4333C>T	(p.Arg1445Ter)	5	DNAH11(NM_001277115.2):c.4942C>T	(p.Gln1648Ter)	4	X	
1-32	DNAI1(NM_012144.4):c.48+2dup	(p.?)	5	DNAI1(NM_012144.4):c.1569G>A	(p.Lys523 = )	4		X
1-39	DNAH11(NM_001277115.2): c.12751_12756del	(p.Val4251_ Lys4252del)	5	DNAH11(NM_001277115.2):c.852_854del	(p.Arg285del)	4	X	
1-47	FOXJ1(NM_001454.4):c.868_871dup	(p.Thr291LysfsTer12)	5				X	
1-49	DNAAF1(NM_178452.6):c.329dup	(p.Asp110GlufsTer8)	5	DNAAF1(NM_178452.6):c.572T>G	(p.Leu191Arg)	5		X
1-52	CFAP300(NM_032930.3): c.198_200delTTTinsCC	(p.Phe67ProfsTer10)	5	CFAP300(NM_032930.3): c.198_200delTTTinsCC	(p.Phe67ProfsTer10)	5		X
1-57	ZMYND10(NM_015896.4):c.47T>G	(p.Val16Gly)	5	ZMYND10(NM_015896.4):c.47T>G	(p.Val16Gly)	5		X
1-74	HYDIN(NM_001270974.2):c.6140C>G	(p.Ser2047Ter)	5	HYDIN(NM_001270974.2):c.6140C>G	(p.Ser2047Ter)	5	X	
1-77	DNAAF6(NM_173494.2):400 kb deletion	(p.?)	5					X
1-83	DNAI1(NM_012144.4):c.48+2dup	(p.?)	5	DNAI1(NM_012144.4):c.48+2dup	(p.?)	5		X
1-91	ODAD2(NM_018076.5):c.2976del	(p.Asp993ThrfsTer14)	4	ODAD2(NM_018076.5):c.2976del	(p.Asp993ThrfsTer14)	4		X
1-93	ODAD1(NM_144577.4):c.226C>T	(p.Gln76Ter)	4	ODAD1(NM_144577.4):c.226C>T	(p.Gln76Ter)	4		X
1-96	DNAH11(NM_001277115.2):c.8719C>T	(p.Pro2907Ser)	5	DNAH11(NM_001277115.2):c.8719C>T	(p.Pro2907Ser)	5	X	
1-100	ODAD2(NM_018076.5):c.2528dup	(p.Leu843PhefsTer52)	5	ODAD2(NM_018076.5):c.2528dup	(p.Leu843PhefsTer52)	5		X
1-106	DNAH5(NM_001369.3):c.5563dup	(p.Ile1855AsnfsTer6)	5	DNAH5(NM_001369.3):c.5066T>A	(p.Leu1689Ter)	5		X
1-108	HYDIN(NM_001270974.2):c.6140C>G	(p.Ser2047Ter)	5	HYDIN(NM_001270974.2):c.6140C>G	(p.Ser2047Ter)	5	X	
1-109	DNAH5(NM_001369.3):c.10815del	(p.Pro3606HisfsTer23)	5	DNAH5(NM_001369.3):c.13486C>T	(p.Arg4496Ter)	5		X
1-113	CFAP300(NM_032930.3): c.198_200delTTTinsCC	(p.Phe67ProfsTer10)	5	CFAP300(NM_032930.3):c.353A>G	(p.Asp118Gly)	4		X
1-115	DNAH5(NM_001369.3):c.10815del	(p.Pro3606HisfsTer23)	5	DNAH5(NM_001369.3):c.10615C>T	(p.Arg3539Cys)	4		X
1-116	DNAAF1(NM_178452.6):c.871dup	(p.Ala291GlyfsTer6)	5	DNAAF1(NM_178452.6):c.871dup	(p.Ala291GlyfsTer6)	5		X
1-121	RSPH4A(NM_001010892.3):c.1105G>C	(p.Ala369Pro)	5	RSPH4A(NM_001010892.3):c.1105G>C	(p.Ala369Pro)	5	X	
1-123	DNAI1(NM_012144.4):c.48+2dup	(p.?)	5	DNAI1(NM_012144.4):c.180G>A	(p.?)	4		X
1-128	DNAH5(NM_001369.3):c.5177T>C	(p.Leu1726Pro)	5	DNAH5(NM_001369.3):c.885dup	(p.Lys296GlnfsTer3)	5		X
1-134	CCDC39(NM_181426.2):c.610–2A>G	(p.?)	5	CCDC39(NM_181426.2):c.610–2A>G	(p.?)	5		X
1-135	ZMYND10(NM_015896.4):c.47T>G	(p.Val16Gly)	5	ZMYND10(NM_015896.4):c.47T>G	(p.Val16Gly)	5		X
1-144	DNAH11(NM_001277115.2):c.5506C>T	(p.Arg1836Ter)	4	DNAH11(NM_001277115.2): c.13065_13067del	(p.Leu4356del)	4	X	
1-148	RSPH1(NM_080860.4):c.680dup	(p.Pro228AlafsTer15)	5	RSPH1(NM_080860.4):c.680dup	(p.Pro228AlafsTer15)	5	X	
1-151	DNAH5(NM_001369.3):c.10384C>T	(p.Gln3462Ter)	5	DNAH5(NM_001369.3): Duplication Exon 54-70	(p.?)	5		X
1-153	CCDC40(NM_017950.4):c.248del	(p.Ala83ValfsTer84)	5	CCDC40(NM_017950.4):c.248del	(p.Ala83ValfsTer84)	5		X
1-154	SPAG1(NM_172218.3):c.427–2A>G	(p.?)	4	SPAG1(NM_172218.3):c.595 + 2T>G	(p.?)	4		X
1-171	DNAAF6(NM_173494.2):c.355C>T	(p.Gln119Ter)	5					X
1-179	DNAH5(NM_001369.3):c.12705 + 1G>T	(p.?)	5	DNAH5(NM_001369.3):c.10615C>T	(p.Arg3539Cys)	4		X
1-190	DNAH11(NM_001277115.2): c.12597dup	(p.Pro4200SerfsTer15)	5	DNAH11(NM_001277115.2): c.13420C>T	(p.Gln4474Ter)	4	X	
1-193	DNAH5(NM_001369.3):c.12279 + 1G>A	(p.?)	4	DNAH5(NM_001369.3):c.5177T>C	(p.Leu1726Pro)	5		X
1-194	RSPH4A(NM_001010892.3):c.1391G>A	(p.Gly464Glu)	4	RSPH4A(NM_001010892.3):c.1391G>A	(p.Gly464Glu)	4	X	
1-205	DNAH9(NM_001372.4):c.5106T>G	(p.Tyr1702Ter)	4	DNAH9(NM_001372.4):c.9211_9214dup	(p.Gly3072GlufsTer8)	5	X	
1-207	CCDC40(NM_017950.4):c.940-1G>C	(p.?)	5	CCDC40(NM_017950.4):c.940-1G>C	(p.?)	5		X
1-211	CCDC40(NM_017950.4):c.2440C>T	(p.Arg814Ter)	5	CCDC40(NM_017950.4):c.2440C>T	(p.Arg814Ter)	5		X
1-214	DNAH11(NM_001277115.2): c.11663G>A	(p.Arg3888His)	4	DNAH11(NM_001277115.2): c.11663G>A	(p.Arg3888His)	4	X	
1-218	DNAAF4(NM_130810.4):c.583del	(p.Ile195Ter)	5	DNAAF4(NM_130810.4):c.583del	(p.Ile195Ter)	5		X
1-220	DNAH5(NM_001369.3):c.1715T>G	(p.Leu572Trp)	4	DNAH5(NM_001369.3):c.5146C>T	(p.Arg1716Trp)	5		X
1-221	CCNO(NM_021147.5):c.926del	(p.Pro309ArgfsTer18)	5	CCNO(NM_021147.5):c.926del	(p.Pro309ArgfsTer18)	5	X	
1-226	DNAAF11(NM_012472.6):c.630del	(p.Trp210CysfsTer12)	5	DNAAF11(NM_012472.6):c.630del	(p.Trp210CysfsTer12)	5		X
1-228	DNAAF4(NM_130810.4):c.583del	(p.Ile195Ter)	5	DNAAF4(NM_130810.4):c.808C>T	(p.Arg270Ter)	5		X
1-232	DNAAF11(NM_012472.6):c.630del	(p.Trp210CysfsTer12)	5	DNAAF11(NM_012472.6):c.630del	(p.Trp210CysfsTer12)	5		X
1-233	ODAD4(NM_001350319.2):c.245del	(p.Lys82ArgfsTer29)	5	ODAD4(NM_001350319.2):c.397 + 1G>A	(p.?)	5		X
1-238	CCDC40(NM_017950.4):c.248del	(p.Ala83ValfsTer84)	5	CCDC40(NM_017950.4):c.736_755dup	(p.Ser252ArgfsTer43)	4		X
1-242	ODAD1(NM_144577.4):c.742G>A	(p.Ala248Thr)	5	ODAD1(NM_144577.4):c.742G>A	(p.Ala248Thr)	5		X
1-255	DNAH5(NM_001369.3):c.2710G>T	(p.Glu904Ter)	5	DNAH5(NM_001369.3):c.2710G>T	(p.Glu904Ter)	5		X
1-258	RSPH4A(NM_001010892.3): c.1963_1966del	(p.Asp655IlefsTer83)	4	RSPH4A(NM_001010892.3): c.1963_1966del	(p.Asp655IlefsTer83)	4	X	
1-261	DNAAF1(NM_178452.6):c.1349dup	(p.Pro451AlafsTer6)	5	DNAAF1(NM_178452.6):c.1349dup	(p.Pro451AlafsTer6)	5		X
1-265	SPAG1(NM_172218.3): c.1282_1294del	(p.Ala428ProfsTer17)	5	SPAG1(NM_172218.3):c.1282_1294del	(p.Ala428ProfsTer17)	5		X
1-292	SPEF2(NM_024867.4):c.910C>T	(p.Arg304Ter)	5	SPEF2(NM_024867.4):c.2629del	(p.Ile877PhefsTer6)	4	X	

CRS was diagnosed in accordance with the EPOS guidelines (Fokkens et al., 2020). Basic clinical information was collected from the International PCD registry (Werner et al., 2016). HrQoL was evaluated by using the German-adapted 20 item Sino-Nasal Outcome Test (SNOT-20-GAV) (Baumann et al., 2007), and radiologic staging of chronic rhinosinusitis was determined using Lund–Mackay scores (LMS) (Lund and Mackay, 1993).

### 2.2 Genetic analyses

Genetic diagnoses were established using regular gene testing including Sanger sequencing of PCD genes. In most cases, targeted PCD gene panels were used as previously described (Raidt et al., 2022; Aprea et al., 2023). In a few cases, whole-exome sequencing was performed, and data were analyzed only for DNA variants in previously published PCD genes. Only individuals with pathogenic autosomal-recessive bi-allelic variants or a pathogenic heterozygous dominant or hemizygous X-linked variant were included. Segregation analysis was performed when parental DNA was available. All DNA variants were evaluated according to the guidelines of the American College of Medical Genetics and Genomics and the Association for Molecular Pathology (ACMG/AMP) (Richards et al., 2015), and only pathogenic (class 5)/likely pathogenic variants (class 4) were included. The pathogenicity of genetic variants was determined as previously described using *in silico* calculation programs (e.g., Varsome) (Raidt et al., 2022; Aprea et al., 2023). Gene nomenclature was used according to the current approved HGNC [human genome organization (HUGO); (https://www.genenames.org/)] (Bruford et al., 2020).

### 2.3 Imaging analyses

We analyzed available CT and MRI scans of the paranasal sinuses stored in the Picture Archiving and Communication System (PACS) of the University Hospital Muenster. CT scans were assessed in bone windows in axial and coronal slices, while T1-and T2-weighted coronal and axial slices were analyzed from MRI scans as described previously (Lin and Bhattacharyya, 2009). MRI was acquired on different machines with a magnetic field strength of 1.5 or 3.0 Tesla, and spin-echo sequences were used. The slice thickness varied between 3 and 5 mm. Pneumatization of the paranasal sinuses is linked to age, especially of the frontal and sphenoid sinuses (Lee et al., 2022). The most recent imaging was evaluated for statistical analyses.

Scans were evaluated for mucosal thickening and opacification of each of the sinuses and the ostiomeatal complex according to the Lund–Mackay scoring system (0 = no opacification/mucosal swelling; 1 = partial opacification; 2 = complete opacification). Ostiomeatal complexes were either scored as 0 (not obstructed) or 2 points (obstructed) (Hopkins et al., 2007). Agenesis of sinuses was scored with 0 points. Single values were summed, leading to a score between 0 (indicating no opacification at all) and 24 (indicating full opacification of all scored sites). Scoring was performed independently and blinded by both a pediatrician (AS) and a rhinologist (AGB).

Agenesis of the frontal sinus was defined as the absence of supraorbital pneumatization of the frontal bone. Agenesis of the sphenoid sinus was defined as the absence of pneumatization of the sphenoid bone. Paranasal sinus agenesis was only evaluated in individuals aged 16 years or older.

### 2.4 Health-related quality of life

HrQoL was evaluated by using the SNOT-20-GAV, a validated and slightly adapted German translation of the SNOT-20 (Piccirillo et al., 2002; Baumann et al., 2008). The questionnaire contains 20 items addressing upper airway and general symptoms. Compared to the SNOT-20, the SNOT-20-GAV replaces two items addressing night sleep with items addressing CRS symptoms (nasal congestion and reduced sense of smell) (Baumann, 2009). Subscores were calculated as suggested by Baumann et al., (2008). Individuals with PCD and of at least 18 years of age answered the questionnaire during routine consultations in the outpatient clinic between June 2017 and October 2020. SNOT-20-GAV scores were correlated with LMS, where paired data were available.

### 2.5 Statistics

Statistics were mainly calculated with Graphpad Prism 9.2.0 for Windows. Intraclass correlation (ICC), Fisher’s exact test, and chi-squared test were calculated using IBM SPSS Statistics version 28.0.1.0. For LMS and SNOT scores, Gaussian distribution was assumed, and unpaired *t*-test was calculated between ultrastructural groups and sex. Correlation between LMS, SNOT-20-GAV, and their subscores was evaluated by Pearson correlation. Concordance of Lund–Mackay Scores and sinus agenesis assessment was quantified using the intraclass correlation coefficient with a two-way mixed model for absolute agreement on average measures. Dependency of ciliary ultrastructure on agenesis of paranasal sinuses was analyzed by chi-squared test, and relative risks for paranasal sinus surgery between subgroups of predicted ultrastructure were evaluated by Fisher’s exact test. Scatterplots show each data point as individual points in the graph. The horizontal line and error bars indicate mean and standard deviation. The level of significance was determined as alpha = 0.05.

## 3 Results

### 3.1 Study cohort and genetic results

The cohort contained 58 individuals with genetically confirmed PCD. Disease-causing variants were identified in 22 different PCD genes. In most cases, these genetic variants were inherited in an autosomal recessive manner (55/58), but in two individuals a rare X-linked mutation in *DNAAF6* and in one individual an autosomal dominant mutation in *FOXJ1* were reported (Table 1). An overview of the detected genetic defects in this study cohort is summarized in Table 1. There were 26 male and 32 female individuals included. The mean age of the study cohort was 30.9 years (±14.5 SD, range 5–65 years) (Table 2). We characterized the cohort according to the predicted ciliary ultrastructure as indicated by genetic defect. In all, 42 individuals were classified into the group with predicted abnormal ultrastructure (72%), including individuals with pathogenic variants in *CCDC39* (*n* = 1), *CCDC40* (*n* = 4), *CFAP300* (*n* = 2), *DNAAF1* (*n* = 3), *DNAAF4* (*n* = 2), *DNAAF* 6 (*n* = 2), *DNAAF11* (*n* = 2), *DNAH5* (*n* = 10), *DNAI1* (*n* = 4), *ODAD1* (*n* = 3), *ODAD2* (*n* = 2), *ODAD4* (*n* = 1), *SPAG1* (*n* = 2), and *ZMYND10* (*n* = 4). Meanwhile, 16 individuals were classified into the subgroup with predicted (near) normal ultrastructure (28%), including individuals with pathogenic variants in *CCNO* (*n* = 1), *DNAH11* (*n* = 6), *DNAH9* (*n* = 1), *FOXJ1* (*n* = 1), *HYDIN* (*n* = 2), *RSPH1* (*n* = 1), *RSPH4A* (*n* = 3), and *SPEF2* (*n* = 1) (Table1). All individuals in this cohort suffered from chronic rhinosinusitis (100%). Moreover, 27 individuals (47%) underwent sinus surgery at least once, 7 of whom underwent multiple surgeries (26%) (Table 2). There was no difference in relative risk for paranasal sinus surgery between the groups of predicted abnormal compared to predicted (near) normal ultrastructure (relative risk 0.77; *p* = 0.392).

**TABLE 2:** Clinical characteristics of the PCD cohort Shown are ratios, mean ± standard deviation (minimum—maximum), and counts (percentages). Abbreviations: PCD, primary ciliary dyskinesia; CT, computed tomography; MRI, magnetic resonance imaging; SNOT, sino-nasal outcome test; m, male; f, female.

Characteristics	PCD individuals *n* = 58	Sinus imaging n = 36, CT = 25, MRI = 11	SNOT *n* = 36
Sex (m:f)	26:32	14:22	14:22
Age in years	30.9 ± 14.5 (5—65)	24.3 ± 10,2 (5–48)	36.6 ± 13.7 (20—65)
Age on imaging/SNOT		18.9 ± 9.2 (4—43)	31.7 ± 13.2 (18—63)
Predicted ciliary ultrastructure (near) normal:abnormal)	16:42	9:27	12:24
Chronic rhinosinusitis [(near) normal:abnormal)]	58 (100%) [16:42]	36 (100%) [9:27]	36 (100%) [12:24]
Paranasal sinus surgery [(near) normal:abnormal)]	27 (47%) [9:18]	18 (50%) [5:13]	16 (44%) [5:11]
Resurgery [(near) normal:abnormal)]	7/27 (26%) [3:4]	5/18 (28%) [3:2]	4/15 (25%) [1:3]

### 3.2 Imaging findings

Paranasal sinus CT (*n* = 25) and MRI (*n* = 11) scans were available from 36 individuals. The mean age at the time of imaging was 18.9 ± 9.2 years (range 4—43). When comparing CT and MRI scans in this cohort, there was no difference in LMS between these groups (CT 10.4 ± 4.3, MRI 9.9 ± 3.6, *p* = 0.74).

**FIGURE 1:**
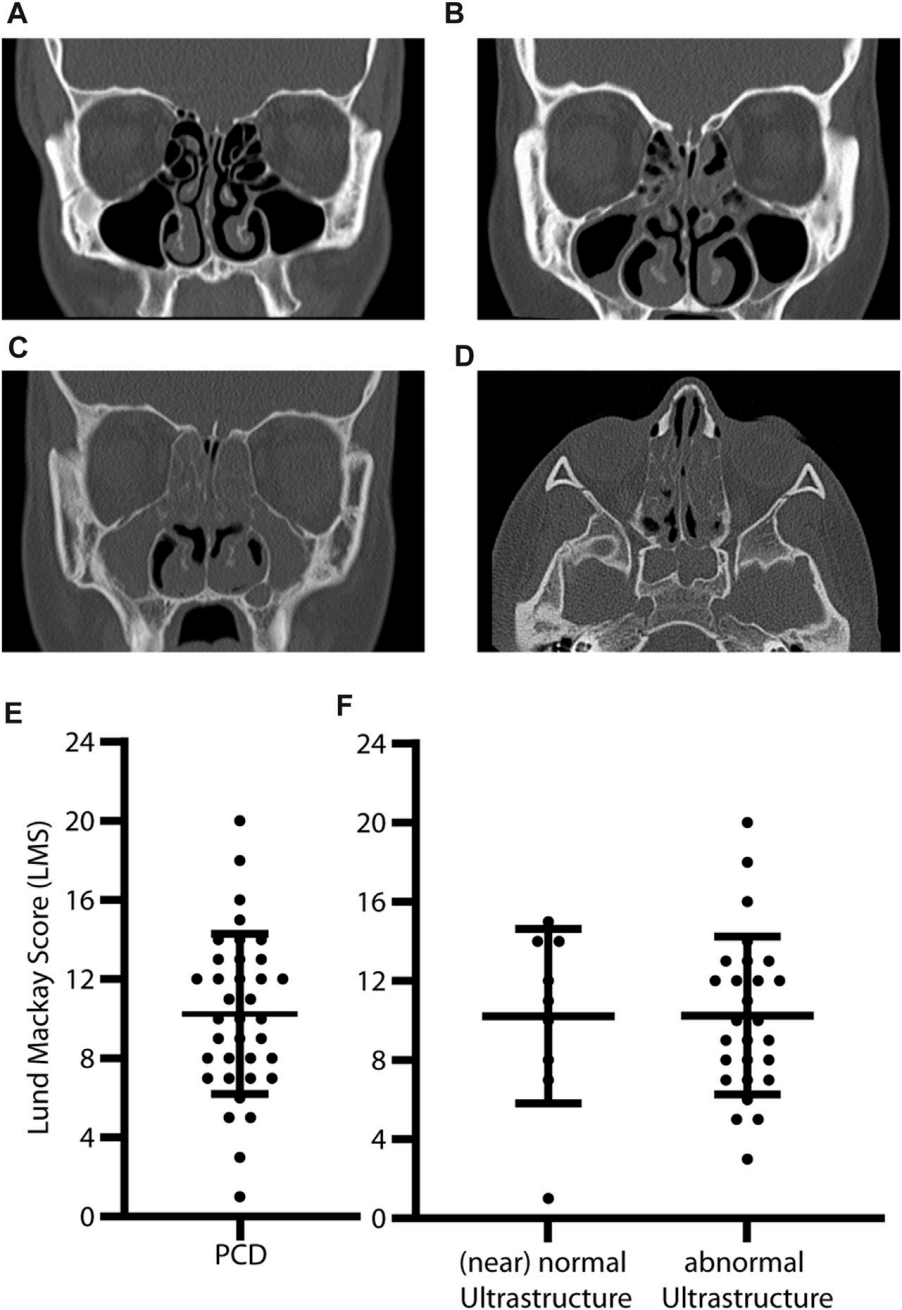
Abnormal opacification of paranasal sinuses and high Lund–Mackay Scores in PCD individuals. Coronal **(A–C)** and axial **(D)** CT slices of individuals with PCD show **(A)** no opacification, **(B)** partial opacification, and **(C)** full opacification of the maxillary sinuses (asterisk) and ethmoid cells (arrowhead). Chronic rhinosinusitis can lead to osteogenesis of ethmoid cells and medial orbital walls **(C,D)**. Lund–Mackay Scores are calculated by grading six different sites on each side with 0-2 points (0 = no, 1 = partial, and 2 = full opacification). The most severe score is 24. In this study, individuals with PCD have moderate-to-severe opacification with a mean LMS of 10.3 ± 4 **(E)**. There is no difference between PCD with predicted (near) normal (mean LMS 10.2 ± 4.4) and abnormal (mean LMS 10.3 ± 4.4) ciliary ultrastructure based on genetic defects **(F)** (*p* = 0.98). Error bars indicate mean ± SD.

**FIGURE 2:**
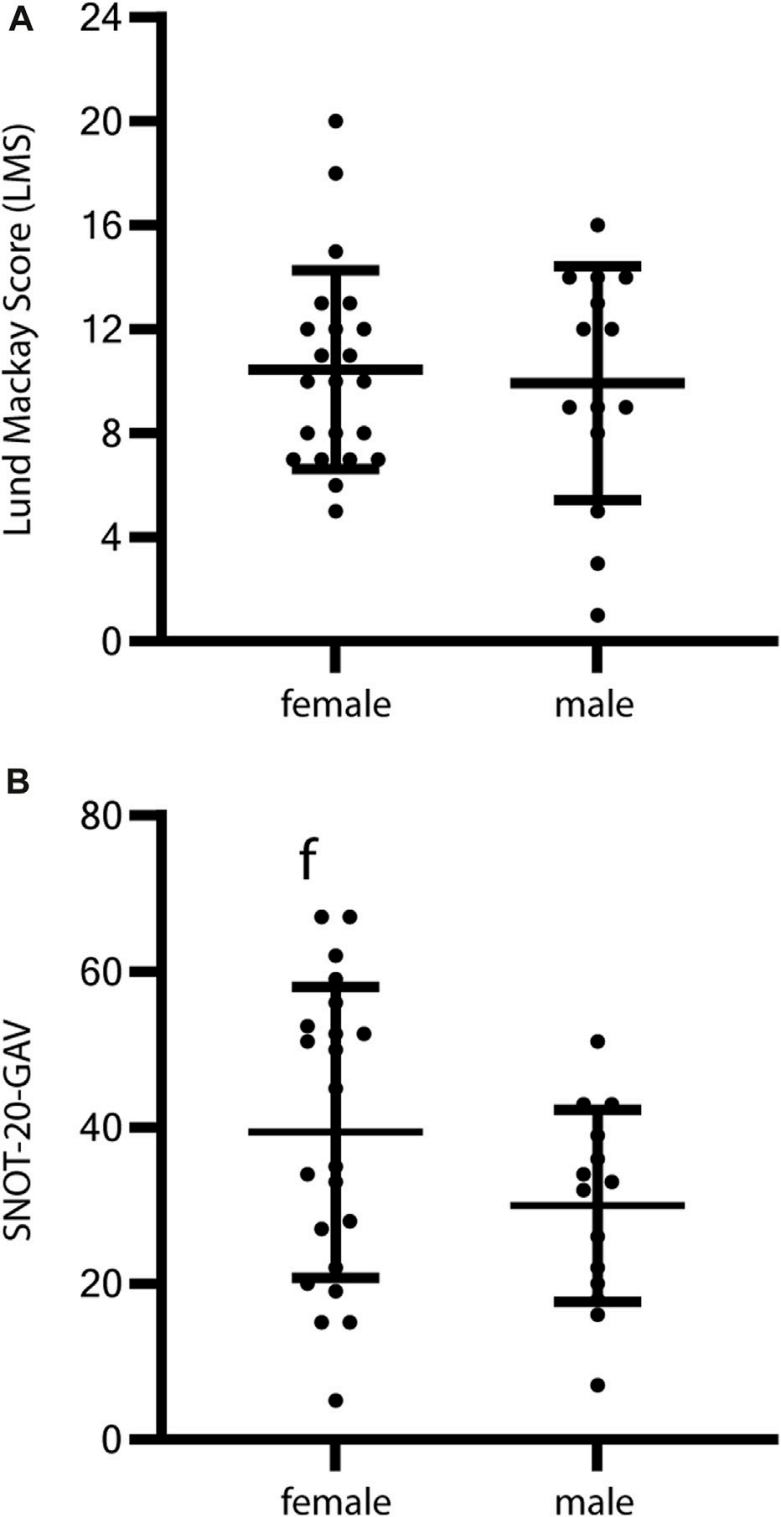
Sex-specific analysis of HrQoL and radiologic grading of CRS in PCD individuals. Evaluation of HrQoL using SNOT-20-GAV and radiologic grading by Lund–Mackay Score (LMS) in relation to sex show no significant differences. **(A)** Radiologic grading for female PCD individuals shows a mean LMS of 10.5 ± 3.8 and for male PCD individuals of 9.9 ± 4.5 (*p* = 0.71). **(B)** The HrQoL shows a slight tendency toward a reduced HrQoL in female individuals (mean SNOT-20-GAV 39.4 ± 3.8) compared to male individuals (mean SNOT-20-GAV 30 ± 12.3), but the difference does not reach statistical significance (*p* = 0.11). Error bars indicate mean ± SD.

The amount of opacification was graded (0 = no opacification, 1 = partial opacification, 2 = complete opacification) from imaging for each paranasal sinus and ostiomeatal complex on each side (12 sites) according to the Lund–Mackay score (Lund and Mackay, 1993). The mean LMS was 10.3 ± 4.0, indicating moderate-to-severe opacification (Figure 1). There was no significant difference in radiologic grading (*p* = 0.98) between the PCD subgroups with predicted (near) normal and abnormal ultrastructure (Figure 1F) and between sexes (*p* = 0.71, Figure 2A).

**FIGURE 3:**
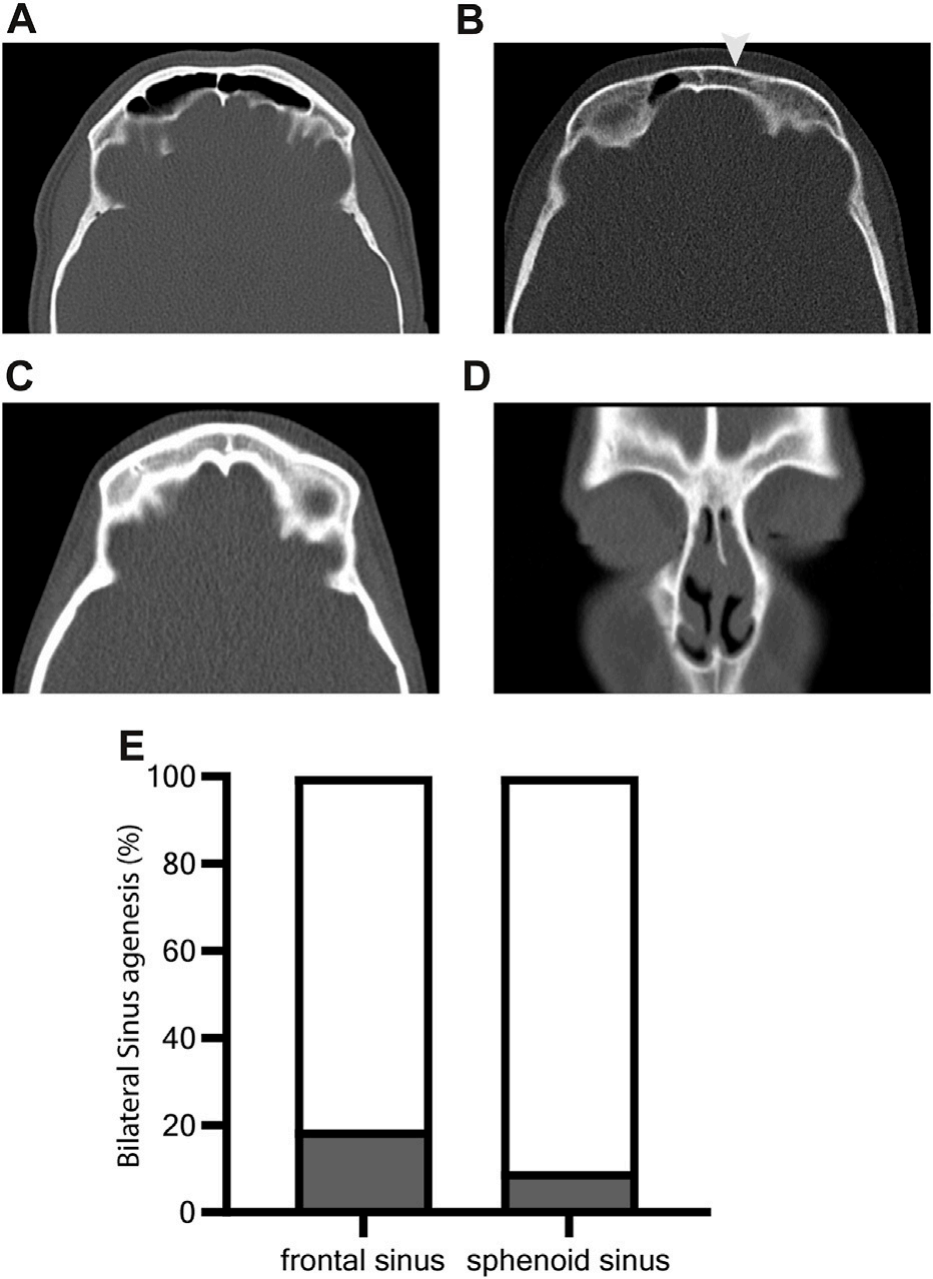
Increased prevalence of frontal and sphenoid sinus agenesis in PCD. Supraorbital axial **(A–C)** and ventral coronal **(D)** CT slices of individuals with PCD show **(A)** normal developed frontal sinus, **(B)** unilateral agenesis (arrowhead) of frontal sinus, and **(C,D)** bilateral agenesis of sinus frontalis. Paranasal sinus aplasia was investigated for individuals aged 16 years or older as the pneumatization of paranasal sinuses develops during adolescence The prevalence of bilateral agenesis of the frontal sinus is 19% (4/21). Bilateral agenesis of the sphenoid sinus occurs less often (9.5%, 2/19) **(E)**.

Analysis of individuals aged 16 years or older (Figure 3) demonstrated a high prevalence of bilateral agenesis of the frontal (4/21, 19%) and sphenoid sinuses (2/21, 9.5%). Genetic defects resulting in agenesis of the frontal (*CCNO*, *DNAAF1*, *DNAH11*, and *HYDIN*) and sphenoid (*HYDIN* and *CCDC40*) sinuses comprise genetic defects that are predicted to result in both PCD with (near) normal and abnormal ultrastructure.

### 3.3 Quality of life assessment

**FIGURE 4:**
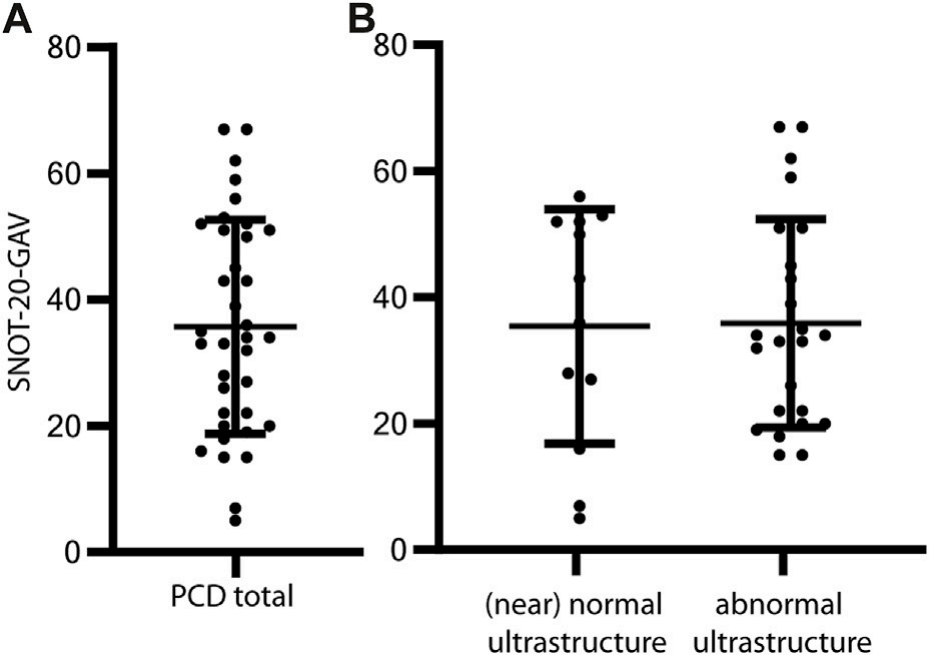
SNOT-20-GAV demonstrates high disease burden associated with CRS in PCD. The German-adapted version of the 20-item sino-nasal outcome test (SNOT-20-GAV) was obtained from PCD individuals during routine consultations in the outpatient clinic. The items address chronic rhinosinusitis (CRS) symptoms such as nasal drip and general aspects such as fatigue. Participants rate each item from zero points (no problem) to five points (problem as bad as it can be). The total score ranges from 0 to 100 points, with higher scores indicating a reduced quality of life. On average, individuals with PCD score 35.1 ± 17 points in SNOT-20-GAV **(A)**. The difference between PCD with predicted (near) normal (mean SNOT-20-GAV 36.1 ± 17.3) and predicted abnormal ciliary ultrastructure (mean SNOT-20-GAV 34.7 ± 17.2) does not reach statistical significance (*p* = 0.83) **(B)**. Error bars indicate mean ± SD.

HrQoL was assessed with the standardized questionnaire SNOT-20-GAV that was answered by a total of 36 patients during routine visits at outpatient clinics. The mean SNOT-20-GAV was 35.8 ± 17 (Figure 4). This indicates a substantial reduction in HrQoL. Subscores were highest for primary nasal symptoms (44 ± 19), followed by secondary rhinogenic symptoms (33.4 ± 17.6) and general items concerning quality of life (32.7 ± 19.8). Cough, congested nose breathing, runny nose, were the complaints with the highest scores (mean 2.6—3.2) in SNOT-20-GAV. Ear pain, dizziness, facial pain/pressure, and sneezing were the least common (mean 0.66–0.83). HrQoL showed a slight tendency toward a reduced HrQoL in female individuals (mean SNOT-20-GAV ±SD 39.4 ± 3.8) compared to male individuals (mean SNOT-20-GAV ±SD 30 ± 12.3), but the difference did not reach statistical significance (*p* = 0.11, Figure 2B). The correlation between LMS and SNOT-20-GAV (Pearson r = 0.35) was weak and did not reach statistical significance. There was no significant difference HrQoL (*p* = 0.93) between the predicted (near) normal and abnormal ultrastructure groups (Figure 4).

## 4 Discussion

Reports on CRS in individuals with PCD are scarce. Here, we investigated the clinical phenotype of the upper airways with a focus on the paranasal sinuses in a large cohort of 58 individuals with genetically confirmed PCD. Our cohort comprised PCD individuals with disease-causing variants in 22 different genes illustrating the high genetic heterogeneity of PCD (Wallmeier et al., 2020; Pennekamp et al., 2023). We investigated the impact of CRS based on radiologic imaging and the Lund–Mackay scoring system (LMS) and health-related quality of life (HrQoL) to grade CRS severity.

In this study, we decided not to modify LMS for the absence of sinuses compared to other investigations (Pifferi et al., 2011; Berkhout et al., 2016). The modification would increase the total scores in case of sinus aplasia, implicating a higher disease severity. However, aplastic sinuses cannot become inflamed, and a modification of LMS would therefore distort the results. In addition, available sinus imaging showed a moderate-to-severe grade of opacification of sinuses, as indicated by a mean LMS of 10.3. An LMS of four or higher in adolescents and five or higher in children is considered pathologic and highly predictive for the diagnosis of CRS if applied using a CT scan (Fokkens et al., 2020). Our cohort was evaluated by both CT and MRI. Based on previous literature, Lund–Mackay scores based on MRI scans do not overestimate sinus opacification (Lin and Bhattacharyya, 2009). This is supported by our findings. When comparing CT and MRI scans in this cohort, there was no difference in LMS between these groups (CT 10.4 ± 4.3, MRI 9.9 ± 3.6, *p* = 0.74). Thus, MRI can be used for future studies in order to reduce the radiation exposure.

The pneumatization of paranasal sinuses develops during adolescence (Barghouth et al., 2002) and is therefore strictly related to the age of individuals. Hence, we investigated paranasal sinus aplasia only in individuals aged 16 years or older. Increased frequency of aplasia of frontal and sphenoid sinus in PCD was also reported previously (Pifferi et al., 2011; Bequignon et al., 2019a; Bhatt et al., 2019). In our study, the frontal sinus was bilaterally aplastic in 19% of individuals (16 years of age or older), and the sphenoid sinus was aplastic in 9.5% of this cohort. This is more frequent than in the general European population, in whom frontal sinus aplasia occurs in only 5% of individuals (Butaric et al., 2020). We demonstrate that frontal sinus aplasia can occur in different genotypes without being prevalent in distinct genotypes or subgroups. The sample size of the subgroups for individual genes was too small for statistical analysis. Further studies with larger samples are needed to uncover potential genotype–phenotype correlations.

Sinus aplasia is not unique to PCD as individuals with cystic fibrosis (CF) suffering from reduced mucociliary clearance, due to abnormally viscous mucus, show a high prevalence as well (Orlandi and Wiggins, 2009; Berkhout et al., 2016; Kang et al., 2017; Halderman et al., 2019). Thus, our data indicate that clinicians who examine patients with severe CRS and underdeveloped paranasal sinuses should consider a disease with reduced mucociliary clearance and, accordingly, refer them to a center specialized in CF and PCD diagnostics, especially when chronic respiratory symptoms are present.

CRS had a strong clinical impact in our cohort as at least 47% of the individuals with PCD underwent sinus surgery once or multiple times (Table 2). This rate might even be underestimated due to recall bias as many adult participants were included who primarily reported pulmonary symptoms. To reduce this bias, we analyzed the imaging for signs of functional endoscopic sinus surgery (FESS). We found seven additional individuals exhibiting signs of FESS who were not reported in electronic health records. Thus, the majority of the individuals with PCD underwent sinus surgery.

The CRS-related QoL was strongly reduced, as indicated by the mean SNOT-20-GAV score of 35. This is considerably higher than the impact of CRS in the healthy general population (a SNOT-20-GAV of 13 is expected) (Baumann et al., 2008) but comparable to the impact of CRS in individuals with CF (mean SNOT-22 of >30) (Habib et al., 2015; Kang et al., 2017) and the impact of primary CRS (Baumann et al., 2008). Interestingly, some individuals reached even less than 5 points in the questionnaire, emphasizing the broad spectrum of disease severity. For CRS, it has been previously reported that female people show a significant reduction in HrQoL (Fokkens et al., 2020). Therefore, we analyzed our HrQoL-Data for sex-specific differences. We could not show any significant sex-specific differences. However, there was a tendency toward reduced HrQoL in female PCD individuals. Therefore, future studies with larger patient cohorts should address this question again.

We also consider certain limitations to this study. First, HrQoL was evaluated with SNOT-20-GAV rather than the highly regarded SNOT 22 (Fokkens et al., 2020; Albrecht et al., 2021). At the time of the initiation of this investigation, SNOT-20-GAV was the only available validated German translation (Baumann, 2009; Albrecht et al., 2021). Due to the recent implementation of SNOT-22 in German (Albrecht et al., 2021), we aim to use a comparable questionnaire for future meta-analyses.

Here, we further evaluated the upper airway symptoms in the genetically defined PCD cohort by grouping PCD individuals based on genetic defects that are predicted to cause either (near) normal (28%) or abnormal (72%) ciliary ultrastructure. Previous studies have shown that there are differences in diagnostic and clinical findings between PCD with abnormal and PCD with (near) normal ultrastructure (Raidt et al., 2022; Kinghorn et al., 2023). Previously, we have shown that PCD individuals with (near) normal ultrastructure have a higher nasal NO production rate, more residual ciliary activity, and lower frequency of laterality defects (Raidt et al., 2022; Pennekamp et al., 2023). We therefore studied if there are also differences regarding upper airway disease.

Interestingly, our analyses did not find evidence for differences in the CRS disease severity among those PCD subgroups because the HrQoL and LMS scores did not differ significantly (Figures 1, 3). Further subgroup analyses were not performed because of the marked genetic heterogeneity present in our PCD cohort (22 different PCD genes were affected).

Because of the high disease burden of CRS in PCD individuals and the frequent paranasal sinus surgeries, our findings strongly support that PCD individuals should be followed by ENT specialists on a regular basis. Current therapy mainly focuses on secretion management by nasal rinsing, nasal inhalation of hypertonic saline solution, and topical steroids (Bequignon et al., 2019b; Paff et al., 2021). Although FESS in PCD individuals showed a positive effect on HrQoL in a small clinical trial with 24 PCD individuals (Alanin et al., 2017), the rates of recurrence and re-intervention are high, as indicated by this investigation. Therefore, surgical interventions should be considered individually based on patient history, physical examination, and reduction of HrQoL (Werner et al., 2015; Fokkens et al., 2020).

In summary, this is the first study to investigate the disease burden of upper airways in a genetically defined cohort of PCD individuals (*n* = 58). As demonstrated by the summary of affected genes in Table 1, a challenge in current PCD investigations is the broad spectrum of different affected genes (Wallmeier et al., 2020) resulting in small subgroups for further genotype–phenotype correlations. Therefore, international collaborative efforts such as the ERNLUNG PCD study group aim to collect ENT data in a systematic way using the international PCD registry (Werner et al., 2016).

## Data Availability

The raw data supporting the conclusion of this article will be made available by the authors, without undue reservation.
